# Factors influencing low sexual desire and sexual distress in pregnancy: A cross-sectional study

**DOI:** 10.18502/ijrm.v19i10.9823

**Published:** 2021-11-04

**Authors:** Mina Malary, Mahmood Moosazadeh, Afsaneh Keramat, Shadi Sabetghadam

**Affiliations:** ^1^Student Research Committee, School of Nursing and Midwifery, Shahroud University of Medical Sciences, Shahroud, Iran.; ^2^Health Sciences Research Center, Faculty of Health, Mazandaran University of Medical Sciences, Sari, Iran.; ^3^Gastrointestinal Cancer Research Center, Non-communicable Diseases Institute, Mazandaran University of Medical Sciences, Sari, Iran.; ^4^Department of Reproductive Health and Midwifery, School of Nursing and Midwifery, Shahroud University of Medical Sciences, Shahroud, Iran.

**Keywords:** Pregnancy, Sexual desire, Sexual distress, Sexual dysfunctions, Influencing factors.

## Abstract

**Background:**

Sexual desire and sexual distress are determined by emotional, psychosocial, hormonal, and anatomical factors during pregnancy.

**Objective:**

To identify the factors contributing to female low sexual desire and sexual distress during pregnancy separately and concurrently.

**Materials and Methods:**

Overall, 295 pregnant women were enrolled in this cross-sectional study. Sexual desire and distress were assessed by the sexual interest and desire inventory-female (score ≤ 33.0 indicates low sexual desire) and the female sexual distress scale-revised (score ≥ 11 indicates sexual distress).

**Results:**

56.3% and 17.3% of pregnant women met the clinical cut-off for low sexual desire and sexual distress, respectively. After adjusting for the effect of the confounding variables by logistic regression multivariate analysis, satisfaction with body image before and during pregnancy, frequency of sexual intercourse, and satisfaction with foreplay were found to be significantly associated with low sexual desire. Factors related to sexual distress were similar to those noted for common sexual desire, except for satisfaction with foreplay. Other factors related to sexual distress included increased age, fear of abortion, and pregnancy trimester. Factors linked to concurrent low sexual desire and sexual distress were similar to those found for sexual distress, except for pregnancy trimester.

**Conclusion:**

Low sexual desire and sexual distress are relatively common sexual experiences during pregnancy. Several factors could predict low sexual desire but were not associated with sexual distress, and conversely. Comprehensive attention to all of these factors is essential while screening for sexual health during pregnancy.

## 1. Introduction

Pregnancy is considered the most critical period in a woman's life (1). It induces significant physical, mental, and sexual changes in the female body (2). Physical and psychological changes, as well as cultural, social, and religious factors can influence sexual activity in pregnancy (3).

Reduced sexual enjoyment, coital frequency, desire, and decreased sexual activity commonly occur during pregnancy. Many factors can influence sexual desire during pregnancy, including living conditions, pregnancy trimester, and physiological alterations (4). Sexual desire is an essential component of romantic relationships, and subsequent sexual activity is an opportunity to establish and increase intimacy between couples (5). Low sexual desire can undermine sexual intimacy or receptivity and may lead to reduced coital frequency. By depriving couples of the various advantages of intercourse, including intimacy and pleasure, low sexual desire threatens the romantic bond and can induce conflict, unfaithfulness, or relationship breakdown (5, 6).

Sexual distress is the negative emotions of individuals regarding their sex life and comprises feelings of embarrassment, blame, frustration, anxiety, fear, and anger (7). The presence of significant sexual distress is a key criterion in the diagnostic process of sexual dysfunction and should always be addressed while assessing sexual function (8). European epidemiological studies have demonstrated that 46-65% of women with sexual problems suffer from sexual distress (9, 10), indicating that impairments in sexual function are associated with sexual distress in some women (but not all) (11). Concern about the impact of sexual activity on the fetus and low sexual desire can instigate or promote sexual distress. There have been few studies on sexual distress during pregnancy. The incidence of sexual disorders and concomitant sexual distress is different in various female populations. For instance, 40% of Canadian and American pregnant women were found to experience sexual distress, and 36% of them suffered from sexual problems. More specifically, 14% of the American patients suffered from sexual distress in the absence of sexual problems, 14% experienced sexual problems in the absence of sexual distress, and 26% were diagnosed with sexual problems with concomitant sexual distress (12).

Unfortunately, despite the extensive consequences of low sexual desire and sexual distress during pregnancy, it is not always addressed during routine prenatal care. Questions about sexual activity in different trimesters of pregnancy still cause embarrassment in many women and healthcare professionals. Thus, many questions in this context remain unanswered. No study has yet been conducted in Iran to identify the factors associated with low sexual desire and sexual distress among pregnant women. Therefore, we sought to investigate the factors causing common sexual desire and sexual distress in pregnant women separately and concurrently. It is hoped that raising awareness regarding this issue among pregnant women and healthcare professionals can improve screening and early detection of these problems in the early stages of pregnancy.

## 2. Materials and Methods

### Study population and design

This cross-sectional study was performed with 295 pregnant women between September 2019 and January 2020 according to the Strengthening the Reporting of Observational Studies in Epidemiology (STROBE) guidelines in Amol, north of Iran (13).

The participants were selected using the two-stage cluster sampling method. In the first stage, four out of 18 health centers were selected randomly from the north, south, east, and west of Amol city. Afterward, the participants were chosen systematically from each center based on the probability of selection in proportion to the population size (or estimated population size). Those meeting the inclusion criteria were invited to participate in the study.

The inclusion criteria included singleton pregnancy, lack of any diseases, living with the partner at the time of participation, and willingness to participate. The exclusion criteria were psychological or psychiatric comorbidities, any medical illness, contraindication for sexual intercourse, and conceived via assisted reproductive techniques.

### Outcome measurement

Data were gathered using three instruments: a related factors checklist, the sexual interest and desire inventory-female (SIDI-F), and the female sexual distress scale-revised (FSD-R).

The related factors checklist included items on demographic characteristics, obstetrics history, and sexual experience. Demographic characteristics included maternal age, duration of the marriage, level of education, occupation, and satisfaction with income. The obstetrics history section recorded parity, history of abortion and complications in a previous pregnancy, pregnancy trimester, and fear of abortion due to sexual activity in current pregnancy. In the sexual experience section, the following questions were asked: Are you satisfied with foreplay before sexual activity? Is your sexual activity scheduled? How many times have you had sexual intercourse in the previous month? To evaluate the body image (BI) satisfaction, participants were asked to provide their opinion as to how physically attractive they were before and during pregnancy using a 3-point Likert scale ranging from not very attractive (1) to very attractive (3).

In this study, the validated Farsi version of the SIDI-F was employed to evaluate female sexual desire in the previous month (14). The SIDI-F is a 13-item questionnaire rated by a clinician. Items are ranked based on a 5-point scale ranging from 0 to 5, with higher scores indicating greater levels of sexual desire. The minimum and maximum possible scores for this questionnaire are 0 and 51, respectively. The SIDI-F had a Cronbach's alpha of 0.90 and excellent internal consistency. The convergent validity of this instrument was established by correlating its results with those of other valid sexual function instruments. We found that the total score of the SIDI-F was highly associated with the satisfaction, arousal, and desire domains of the FSFI (all correlations > 0.8) (15). The internal consistency reliability of the Farsi version was 0.89, indicating excellent reliability. In addition, test-retest reliability with a 2-wk interval revealed good reliability of this tool (14).

The FSD-R is a self-report instrument used to examine sexual distress during the past month. Each item is rated from never (0) to always (4), with scores ranging from 0 to 52. Women with a score of at least 11 were classified as having sexual distress. This questionnaire was shown to have high internal consistency (Cronbach's alpha = 0.96) and validity (92.7%) (7). The internal consistency and reliability of the Farsi version of FSD-R were calculated to be > 0.70 by Azimi Nekoo and colleagues (16). Therefore, it is considered a valid and reliable tool for assessing sexual distress among Iranian women.

### Sample size calculation

In a study of pregnant women aged 18-40 yr, the mean and standard deviation (SD) of sexual desire as evaluated by the female sexual function index (FSFI) was 1.67 (SD = 1.67) (17). With an estimated accuracy of 20% (d = 0.2) at a two-tailed 5% significance difference (α) (Z = 1.96), the standard sample size was computed to be 267 using the following formula. Considering a 15% attrition rate, the final sample size was calculated at 314. 


$$n=\frac{z^{2}\sigma^{2}}{d^{2}}$$

### Ethical considerations

The necessary scientific approvals were obtained from the Mazandaran University of Medical Sciences, and ethical approval was granted by the Ethics Committee of Shahroud University of Medical Sciences (Code: IR.SHMU.REC.1397.098). Written informed consent was obtained from all participants. Prior to performing the study, the eligible women were informed about the study objectives and assured of the confidentiality of the data.

### Statistical analysis

The collected data were analyzed using the Statistical Package for the Social Sciences (SPSS version 22.0 for Windows; SPSS Inc., Chicago, USA). Descriptive statistics are presented for the demographic characteristics, obstetrics history, and sexual experience. First, the association of low sexual desire, sexual distress, and concurrent low sexual desire and sexual distress with categorical variables was tested using the Chi-square test and Fisher's exact test. Then, multivariable logistic regression models were used to identify the factors associated with low sexual desire, sexual distress, and concurrent low sexual desire and sexual distress. To adjust for the effect of confounding variables, all factors associated with each outcome (i.e., low sexual desire, sexual distress, and concurrent low sexual desire and sexual distress) based on the Chi-square test and Fisher's exact test results at a significance level of < 0.05 were included in the multivariable regression model. The strength of the associations between the dependent and independent variables was determined by odds ratio (OR) and 95% confidence intervals (CI). A p-value < 0.05 was considered statistically significant.

## 3. Results

A total of 314 questionnaires were sent to four centers, and 307 were returned. In total, 12 were excluded due to being incomplete (7) and based on the exclusion criteria (5). The final sample consisted of 295 pregnant women aged 18-40 yr. Based on the SIDI-F cut-off score, 166 (56.3%) women were at risk for low sexual desire (29.2 ± 10.3). The mean FSD-R score of this cohort (mean ± SD) was 5.55 ± 6.56, with 51 pregnant women (17.3%) attaining scores greater than the clinical cut-off score of 11. Among the 166 participants who were found to be at risk for low sexual desire by SIDI-F, 42 (25.3%) scored 11 or higher on FSD-R. In other words, among the 259 participants, 42 (14.2%) were found to obtain a score of 33 or lower on SIDI-F and 11 or higher on FSD-R.

The participants' characteristics and correlations with low sexual desire, sexual distress, and concurrent low sexual desire and sexual distress are shown in Table I. The significant independent variables and the outcomes presented in Table I were entered into a multivariate logistic regression analysis, the findings of which are shown in Table II.

The factors associated with a lower likelihood of low sexual desire were satisfaction with foreplay, high satisfaction with BI before and during pregnancy, and increased coital frequency in the past month.

The factors associated with lower levels of sexual distress were high satisfaction with BI before and during pregnancy, three to four occurrences of sexual intercourse in the past month, and not being afraid of fetal abortion. In contrast, women aged > 30 yr were at greater risk for sexual distress during pregnancy. The pregnancy trimester was associated with sexual distress as well, such that the second trimester was associated with lower levels of sexual distress than the first trimester.

Finally, high satisfaction with BI before and during pregnancy, three to four occurrences of sexual intercourse in the past month, and not being afraid of fetal abortion were protective factors against concurrent low sexual desire and sexual distress. In contrast, increased maternal age was a risk factor.

**Table 1:** Baseline characteristics and correlation with low sexual desire, sexual distress, and concurrent low sexual desire and distress


	**Low sexual desire**			**Sexual distress**			<**Concurrent low sexual desire and distress**		
**Characteristics of participants (n = 295)**	No	Yes	p-value	No	Yes	p-value	No	Yes	p-value
	**Age (yr)**									
** < 25**	51 (17.3)	52 (17.6)	93 (31.5)	10 (3.4)	94 (31.9)	9 (3.1)	
**25-30**	37 (12.5)	54 (18.3)	79 (26.8)	12 (4.1)	79 (26.8)	12 (4.1)	
** > 30**	41 (13.9)	60 (20.3)	0.34	72 (24.4)	29 (9.8)	0.001	80 (27.1)	21 (7.21)	0.04
**Duration of marriage (yr)**									
**0-5**	71 (24.1)	84 (28.5)		135 (45.8)	20 (6.8)		137 (46.4)	18 (6.1)		
** > 5**	58 (19.7)	82 (27.8)	0.44	109 (36.9)	31 (10.5)	0.03	116 (39.3)	24 (8.1)	0.17
**Women's education**									
**Primary/secondary**
school	10 (3.4)	21 (7.1)	25 (8.5)	6 (2.0)	25 (8.5)	6 (2.0)	
**High school**	67 (22.7)	82 (27.8)	123 (41.7)	26 (8.8)	129 (43.7)	20 (6.8)	
**Undergraduate/**
postgraduate	52 (17.6)	63 (21.4)	0.39	96 (32.5)	19 (6.4)	0.93	99 (33.6)	16 (5.4)	0.68
**Women's occupation**									
**Working **	19 (6.4)	25 (8.5)		38 (12.9)	6 (2.0)		38 (12.9)	6 (2.0)		
**Housewife **	110 (37.3)	141 (47.8)	0.93	206 (69.8)	45 (15.3)	0.48	215 (72.9)	36 (12.2)	0.90
**Satisfaction with income**									
**Yes**	102 (34.6)	120 (40.7)	191 (64.7)	31 (10.3)	194 (65.8)	28 (9.5)	
**No**	27 (9.2)	46 (15.7)	0.18	53 (18.1)	20 (6.8)	0.008	59 (20.1)	14 (4.8)	0.16
**Parity**									
**Primiparous**	62 (21.0)	77 (26.1)	118 (40.0)	21 (7.1)	120 (40.7)	19 (6.4)	
**Multiparous**	67 (22.7)	89 (30.2)	0.77	126 (42.7)	30 (10.2)	0.35	133 (45.1)	23 (7.8)	0.79
**Characteristics of participants (n = 295)**	No	Yes		p-value	No		Yes	p-value		No	Yes	p-value
	**History of abortion**									
**Yes**	27 (9.2)	38 (12.9)	52 (17.6)	13 (4.4)	54 (18.3)	11 (3.7)	
**No**	102 (34.6)	128 (43.4)	0.68	192 (65.1)	38 (12.9)	0.51	199 (67.5)	31 (10.5)	0.48
**Number of children**									
**No children**	72 (24.4)	95 (32.2)	143 (48.5)	24 (8.1)	145 (49.2)	22 (7.5)	
**1**	41 (13.9)	55 (18.6)	72 (24.4)	24 (8.1)	79 (26.8)	17 (5.8)	
** ≥ 2**	16 (5.4)	16 (5.4)	0.74	29 (9.8)	3 (1.0)	0.04	29 (9.8)	3 (1.0)	0.42
**Trimester**									
**First**	18 (6.1)	27 (9.2)		32 (10.8)	13 (4.4)		36 (12.2)	9 (3.1)		
**Second**	62 (21.0)	77 (26.1)	123 (41.7)	16 (5.4)	126 (42.7)	13 (4.4)	
**Third**	49 (16.6)	62 (21.0)	0.85	89 (30.2)	22 (7.5)	0.019	91 (30.8)	20 (6.8)	0.07
**The complication in a previous pregnancy**									
**Yes**	16 (5.4)	16 (5.4)		20 (6.8)	12 (4.1)		25 (8.5)	7 (2.4)		
**No**	135 (38.3)	150 (50.8)	0.44	228 (75.9)	39 (13.2)	0.001	228 (77.3)	35 (11.9)	0.19
**Fear of fetal abortion due to sexual activity**									
**Yes**	57 (19.3)	88 (29.8)	104 (35.3)	41 (13.9)	113 (38.3)	32 (10.8)	
**No**	72 (24.4)	78 (26.4)	0.13	140 (47.5)	10 (3.4)	≤ 0.001	140 (47.5)	10 (3.4)	≤ 0.001
**Planning for sexual activity**									
**Yes**	43 (14.6)	85 (28.8)	100 (33.9)	28 (9.5)	104 (35.3)	24 (8.1)	
**No**	86 (29.2)	81 (27.5)	0.002	144 (48.8)	23 (7.8)	0.06	149 (50.5)	18 (6.1)	0.06
Satisfaction with foreplay*									
**Yes**	3 (1.0)	25 (8.5)	22 (7.5)	6 (2.0)	24 (8.1)	4 (1.4)	
**No**	126 (42.7)	141 (47.8)	≤ 0.001	222 (75.3)	45 (15.3)	0.58	229 (77.6)	38 (12.9)	0.59
**Satisfaction with body image before pregnancy**									
**Low**	11 (3.7)	26 (8.8)	26 (8.8)	11 (3.7)	29 (9.8)	8 (2.7)	
**Moderate**	82 (27.8)	125 (42.4)	170 (57.6)	37 (12.5)	175 (59.3)	32 (10.8)	
**High**	36 (12.2)	15 (5.1)	≤ 0.001	48 (16.3)	3 (1.0)	0.01	49 (16.6)	2 (0.7)	0.04
**Satisfaction with body image during pregnancy**									
**Low**	36 (12.2)	59 (20.0)		70 (23.7)	25 (8.5)		75 (25.4)	20 (6.8)		
**Moderate**	73 (24.7)	98 (33.2)	146 (49.5)	25 (8.5)	150 (50.8)	21 (7.1)	
**High**	20 (6.8)	9 (3.1)	0.01	28 (9.5)	1 (0.3)	0.006	28 (9.5)	1 (0.3)	0.03
**N. sexual intercourses in the past month**									
**Never**	2 (0.7)	41 (13.9)		30 (10.2)	13 (4.4)		32 (10.8)	11 (3.7)		
**1-2 times a month**	33 (11.2)	63 (21.4)	73 (24.7)	23 (7.8)	75 (25.4)	21 (7.1)	
**3-4 times a month**	52 (17.6)	50 (16.9)	96 (32.5)	6 (2.0)	96 (32.5)	6 (2.0)	
**More than once**
a wk	42 (12.2)	12 (4.4)	≤ 0.001	45 (15.3)	9 (3.1)	0.001	50 (16.9)	4 (1.4)	0.001
Data presented as n (%). Chi-square or Fisher's exact tests. ${}^{*}$ Fisher's exact test was used for this variable									

**Table 2:** Logistic regression for predictors of low sexual desire, sexual distress, and concurrent low sexual desire and distress


	Adjusted OR (95% CI)*					
	**Low sexual desire**		**Sexual distress**		<**Concurrent low sexual desire and distress**	
**Independent variable (n = 295)**	**OR (95% CI)**	**p-value**	**OR (95% CI)**	**p-value**	**OR (95% CI)**	**p-value**
	**Age (yr)**	NI		-		-	
	** < 25**	-		1.0 (ref.)		1.0 (ref.)	
**25-30**	-		2.1 (0.6-6.9)	0.20	1.6 (0.57-4.56)	0.36
** > 30**	-		11.7 (3.3-41.1)	≤ 0.001	8.7 (3.2-23.68)	≤ 0.001
**Duration of marriage, yr**	NI		-		NI	
**0-5**	-		1.0 (ref.)		-	
** > 5**	-		0.75 (0.22-2.5)	0.63	-	
**Satisfaction with income**	NI		-		NI	
**Yes**	-		1.0 (ref.)	-	
**No**	-		0.80 (0.32-2.01)	0.66	-	
**Number of children**	NI		-		NI	
**No children**	-		1.0 (ref.)		-	
**1**	-		1.6 (0.51-5.2)	0.39	-	
** ≥ 2**	-		0.29 (0.04-1.9)	0.21	-	
**Trimester**	NI		-		NI	
**First**	-		1.0 (ref.)		-	
**Second**	-		0.18 (0.05-0.58)	0.004	-	
**Third**	-		0.31 (0.11-1.90)	0.07	-	
**The complication in a previous pregnancy**	NI		-		NI	
**Yes**	-		1.0 (ref.)		-	
**No**	-		0.65 (0.19-2.2)	0.49	-	
**Fear of fetal abortion due to sexual activity**	NI		-		-	
**Yes**	-		1.0 (ref.)		1.0 (ref.)	
**No**	-		0.11 (0.04-0.32)	≤ 0.001	0.11 (0.04-0.27)	≤ 0.001
**Planning for sexual activity**	-		NI		NI	
**No**	1.0 (ref.)		-		-	
**Yes**	0.68 (0.37-1.2)	0.24	-		-	
**Satisfaction with foreplay**	-		NI		NI	
**No**	1.0 (ref.)	-		-	
**Yes **	0.17 (0.04-0.6)	0.01	-		-	
**Satisfaction with body image before pregnancy**	-		-		-	
**Low**	1.0 (ref.)		1.0 (ref.)		1.0 (ref.)	
**Moderate**	0.56 (0.22-1.4)	0.27	0.53 (0.14-1.99)	0.35	0.59 (0.18-1.8)	0.37
**High**	0.22 (0.07-0.7)	0.01	0.11 (0.01-0.73)	0.02	0.14 (0.02-0.74)	0.02
	**Low sexual desire**		**Sexual distress**		<**Concurrent low sexual desire and distress**	
**Independent variable (n = 295)**	**OR (95% CI)**	**p-value**	**OR (95% CI)**	**p-value**	**OR (95% CI)**	**p-value**
	**Satisfaction with body image during pregnancy**	-		-		-	
	**Low **	1.0 (ref.)		1.0 (ref.)		1.0 (ref.)	
**Moderate**	1.1 (0.57-2.2)	0.70	0.43 (0.18-1.02)	0.05	0.64 (0.28-1.42)	0.27
**High**	0.19 (0.05-0.6)	0.01	0.05 (0.004-0.71)	0.02	0.09 (0.009-0.95)	0.04
**N. sexual intercourses in the past month**	-		-		-	
**Never**	1.0 (ref.)		1.0 (ref.)		1.0 (ref.)	
**1-2 times a month**	0.07 (0.01-0.32)	0.001	0.92 (0.31-2.7)	0.88	0.98 (0.36-2.61)	0.96
**3-4 times a month**	0.03 (0.007-0.16)	≤ 0.001	0.16 (0.04-0.60)	0.007	0.19 (0.06-0.65)	0.008
**More than once a weak**	0.01 (0.003-0.06)	≤ 0.001	0.81 (0.21-3.08)	0.76	0.75 (0.22-2.45)	0.63
Multivariate logistic regression analysis was used for this table. ${}^{*}$ Adjusted for all other variables in the table unless indicated as NI. CI: Confidence interval, OR: Odds ratio, NI: Not included in the model						

## 4. Discussion

The results showed that 56.3% and 17.3% of pregnant women experienced low sexual desire and sexual distress during pregnancy, respectively. Of the women with low sexual desire (n = 166), 42 (25.3%) suffered from sexual distress. More than half of the pregnant women who had low sexual desire did not suffer from sexual distress (74.7%). Similarly, it was reported that 70% of pregnant women were not concerned about decreased sexual desire during their pregnancy (17). In our study, sexual distress was not observed in most women with low sexual desire during pregnancy. The rate of sexual distress in this study was lower than those found in Canadian and American population-based studies of pregnant women, which reported that 40% of women suffered from sexual distress during pregnancy (12). It seems that the difference in sociodemographic and cultural characteristics, methods of assessment, and the difference in the cut-off points used for determining low sexual desire and sexual distress may explain the different findings.

Satisfaction with foreplay was found to have a positive influence on sexual desire in the current study. In a Finnish study of nonpregnant women, the primary sexual complaint of women with a partner was the duration of foreplay. Too short foreplay was strongly associated with all domains of sexual function (such as orgasm and arousal) except for sexual desire (18). Although no association was reported between foreplay and sexual desire in that study, we found a significant link between satisfaction with foreplay and sexual desire. The effect of this factor on sexual desire seems to be crucial during pregnancy because of the unique characteristics of this period.

The present study demonstrated that a negative perception of physical attractiveness was associated with low sexual desire, sexual distress, and concurrent low sexual desire and sexual distress. The impact of BI on sexual function and sexual distress has been documented in previous research. Lo and colleagues reported that the self-perception of unattractiveness was associated with both sexual problems and distress in young nonpregnant women (19). In addition, the association between sexual function and BI in nonpregnant women was corroborated (20, 21). Specifically, previous investigations have shown that body shape satisfaction predicts sexual desire (22, 23). Negative feelings toward others' evaluation of one's body can predict declined sexual desire as well (22). In a population-based study in Iran, it was shown that nonpregnant women who were dissatisfied with their body image were almost four times more likely to develop hypoactive sexual desire disorder (24). Body size and shape are fundamentally changed during pregnancy (25). Therefore, it is common to encounter BI disturbances in this period (26). The negative perception of BI is found to play an important role in sexual dysfunction during pregnancy (27). However, the findings are contradictory in this regard. For example, contrary to this study, in a study with Iranian pregnant women, it was found that sexual function and BI scores were not related (2). This discrepancy could be attributed to different tools being used to measure BI, sexual desire, and distress. Although a standardized instrument was not used in the present study to evaluate BI, this factor appears to play a significant role in sexual desire and sexual distress during pregnancy.

The present study also showed a significant association between coital frequency in the past month and low sexual desire, sexual distress, and concurrent low sexual desire and sexual distress, which supports the results of previous studies. In addition, this study demonstrated that fear of harming the fetus due to sexual activity negatively influenced sexual distress and concurrent low sexual desire and sexual distress (but not low sexual desire alone). It seems that sexual distress is caused by the fear of miscarriage or harming the fetus. In one study, concern over harming the fetus was the primary cause of reduced sexual function during pregnancy (4). Likewise, this issue was reported in 66.7% of Tunisian women (28). These misconceptions and beliefs can affect sexual activity during pregnancy. Kong and coauthors studied how obstetric and neonatal outcomes can be affected by sexual intercourse during pregnancy (29). They did not find a significant association between the frequency, experiences, and timing of sexual intercourse and obstetric or neonatal outcomes. Taboos and misconceptions are increased by the lack of communication between healthcare professionals and pregnant women about sexuality. Of note, despite the contraindications for sexual intercourse in high-risk pregnant women such as those with hemorrhage, placenta previa, and preterm premature rupture of membranes, sexual intercourse is encouraged during normal pregnancy.

The present study found that increased age is a significant risk factor for sexual distress and concurrent low sexual desire and sexual distress. Although the effect of women's age on low sexual desire and sexual distress has been established in studies of nonpregnant women, its impact in pregnant women has not been studied yet. An Iranian population-based study on reproductive-age women showed that although increased age in women was associated with elevated low sexual desire, it did not lead to sexual distress (30). This discrepancy could be due to the different target groups of the studies (pregnant vs. nonpregnant). One of the possible reasons for a higher prevalence of low sexual desire and sexual distress in women aged > 30 yr could be the increase in obstetric complications, which lead to elevated distress.

Although pregnancy trimester was revealed to be a significant factor contributing to sexual distress in this study, it was not found to be related to low sexual desire or concurrent low sexual desire and sexual distress. Given the conflicting findings reported in other studies regarding sexual desire, the link between sexual distress and pregnancy trimester remains unknown. In accordance with our present findings on sexual desire in each trimester, it was indicated a significant difference in all sexual function domains (except in the domain of sexual desire) in different trimesters (31). Nevertheless, a longitudinal study reported decreased sexual desire in the first trimester in male and female participants, which slightly improved in the second and third trimesters (6). On the other hand, Fuchs et al. observed higher sexual desire scores in the second trimester than the first and third trimesters (1). This discrepancy may be due to the chosen methods and tools used to assess sexual desire.

The feelings of distress due to sexual issues (e.g., guilt and anxiety) have largely been ignored during pregnancy. In addition, factors associated with sexual pain during pregnancy have not been assessed yet. Nevertheless, this study found that the trimester of pregnancy has a notable influence on sexual distress in women due to the psychological and physiological changes in each trimester. Therefore, healthcare providers must ask pregnant women about their feelings of sexual issues in each trimester.

Although many studies have assessed sexual desire and sexual distress, few have evaluated them during pregnancy. In addition, most studies have used the FSFI to assess sexual desire as a domain of sexual functioning. However, in this study, we measured sexual desire using SIDI-F.

This study had several limitations that need to be considered. First, the data were collected using self-reported questionnaires. However, this might not have affected the results because the questionnaires were completed anonymously with no face-to-face contact. Second, we only assessed pregnant women and ignored their partners. Additionally, there are other sexual problems such as those associated with arousal, orgasm, and pain problems which can also affect sexual desire and sexual distress but which were not addressed in this study. The lack of information about women's psychological status was another limitation. Finally, tracking changes in sexual function and sexual distress, satisfaction, and mental status was impossible in this study. To compare these alterations, future longitudinal studies could be useful to assess women and men simultaneously until and even after childbirth.

## 5. Conclusion

According to the current study's findings, low sexual desire and sexual distress are commonly experienced during pregnancy. Some factors, including satisfaction with BI before and during pregnancy, coital frequency, fear of fetal abortion, and increased age were associated with concurrent low sexual desire and sexual distress in pregnant women, while being satisfied with foreplay was only related to low sexual desire and pregnancy trimester was linked with sexual distress. We hope that our findings lead to an increase in early screening of pregnant women's sexual health by healthcare providers, followed by the necessary interventions to resolve sexual problems.

##  Conflict of Interest

The authors declare that there is no conflict of interest.
